# MutMap Reveals a Structural Deletion at the *Chalcone Synthase* Locus Controlling Black Seed Coat in a Gamma-Irradiated Vietnamese Soybean Mutant

**DOI:** 10.3390/genes17070814

**Published:** 2026-07-17

**Authors:** Chung Thi Bao Pham, Lieu Thi Le, Manh Van Nguyen, Thao Duc Le, Hong Thi Anh Le, Nhu Thi Le, Tuyen Thi Minh Vo, Cuong Nguyen, Dat Tien Nguyen, Pooja Bhatnagar-Mathur, Minh Hong Nguyen, Son Lang Vi

**Affiliations:** 1 Agricultural Genetics Institute, Hanoi 122000, Vietnam; baochungagi@gmail.com (C.T.B.P.); lelieu81vn@gmail.com (L.T.L.); nguyenvanmanhagi@gmail.com (M.V.N.); leducthao246@gmail.com (T.D.L.); anhhongagi@gmail.com (H.T.A.L.); lenhu811@gmail.com (N.T.L.); minhtuyenagi@gmail.com (T.T.M.V.); 2 The Information Technology, Vietnam Academy of Science and Technology, Hanoi 10072, Vietnam; ncuong@ioit.ac.vn; 3 LOBI Vietnam Company Limited, Dai Mo, Hanoi 100000, Vietnam; datnguyen@lobi.vn; 4 Plant Breeding & Genetics Section, Joint FAO/IAEA Centre of Nuclear Techniques in Food and Agriculture, International Atomic Energy Agency, Vienna International Centre, P.O. Box 100, 1400 Vienna, Austria; p.mathur@iaea.org; 5 Faculty of Biotechnology, Chemistry and Environmental Engineering, Phenikaa School of Engineering, Phenikaa University, Duong Noi, Hanoi 12116, Vietnam; minh.nguyenhong@phenikaa-uni.edu.vn; 6 Bioresource Research Center, Phenikaa School of Engineering, Phenikaa University, Duong Noi, Hanoi 12116, Vietnam

**Keywords:** black soybean, *CHS* gene, DT26BS, mapping, MutMap

## Abstract

Black soybean is an important functional crop valued for its high anthocyanin content and associated health benefits, attracting increasing interest in plant breeding and mutation-based approaches to enhance its nutritional and agronomic traits. Here, we investigated a DT26 black seed-coat mutant (DT26BS) derived from the Vietnamese cultivar DT26 following gamma irradiation. Genetic analysis of F_2_ populations indicated that the phenotype is controlled by a single recessive mutation, with additional epistatic interactions observed in other genetic backgrounds. MutMap analysis based on whole-genome sequencing of pooled F_2_ individuals identified a candidate region on chromosome 8 corresponding to the *I* locus. A large deletion (~176 kb) was identified in this region, affecting multiple *Chalcone Synthase* (*CHS*) gene repeats, and which may disrupt RNAi-mediated silencing of *CHS*, thereby triggering anthocyanin restoration in the seed coat. PCR-based markers confirmed tight linkage between this deletion and the black seed-coat phenotype. Crosses with elite lines and further selection until F7 generations showed that the mutation has no adverse effects on major agronomic traits and is useful as a donor for developing improved lines with black seed coat, shorter maturity, high yield, and enhanced anthocyanin content for nutritional improvement. These results demonstrate the utility of MutMap for detecting irradiation-induced structural variants and extend its application to a non-reference elite tropical soybean background, providing a useful genetic resource for black soybean breeding.

## 1. Introduction

Soybean (*Glycine max* (L.) Merr.) is one of the world’s most economically important crops, serving as a major source of protein for animal feed, and human consumption and constituting the largest oilseed commodity in global agriculture. Black soybean is unique among soybean types for its anthocyanin-rich black seed coat and contains diverse bioactive compounds associated with antioxidant, anti-aging, anticancer, antibacterial, and antidiabetic activities [1,2,3,4,5,6,7,8]. Increasing consumer demand for functional foods has renewed interest in black soybean because of its high anthocyanin content and associated health benefits, making it an important target for specialty soybean breeding.

Soybean with black seed coat is caused by the accumulation of flavonoids and anthocyanins in the epidermal layer of the seed coat [9]. Anthocyanins in the seed coat of black soybeans have eight types, among which cyanidin-3-O-glucoside, delphinidin 3-O-glucoside, and petunidin 3-O-glucoside are three primary anthocyanins [2,3]. Black seed coat is reportedly controlled by multiple genetic loci, including *I*, *T*, *W1*, *R*, and *O* that interact epistatically to contribute to the control of soybean seed coloration [10]. Three independent loci (*I*, *R*, and *T*) mainly control the process of the biosynthesizing of the pigments that regulate seed-coat colors. The loci *O* and *W1* determine seed-coat color in case of homozygous recessive *ir* or *it* genotypes, respectively. Black seed coat was reported to be controlled by the level of expression of *Chalcone Synthase* genes [11], which plays a significant role in the anthocyanin biosynthesis pathway, distributing anthocyanin and proanthocyanin pigments. A cluster of *Chalcone Synthase* (*CHS*) genes is located at a region of locus *I* on soybean molecular linkage group A2, chromosome 8 [12,13,14]. Reduction in *CHS* mRNAs and CHS activity was detected in soybean genotypes with dominant *I* (fully buff seed coat and hilum) or *i^i^* (buff seed coat, black hilum) alleles while they were fully expressed in the homozygous recessive *i* allele of pigmented seed coats.

Induced mutagenesis and mutation breeding are effective tools for improving agricultural crops. Gamma rays are a highly energetic form of ionizing radiation capable of causing DNA damage across the entire genome, thereby creating a broad spectrum of genetic variation that can be exploited in plant breeding to develop improved crop varieties [15]. In soybean, gamma rays have been used to create a mutation population with diverse lesions and phenotypes [16] and to create soybean with higher oil and protein content [17].

MutMap is a rapid mapping strategy that integrates bulked segregant analysis with next-generation whole-genome sequencing to identify causal mutations by comparing pooled mutant progeny with the wild-type genome [18,19]. This approach has been successfully applied in crop plants for rapid identification of candidate gene responsible for relevant traits [18,20,21,22]. However, most applications have focused on EMS-induced mutant populations, where dense single-nucleotide polymorphism markers created by chemical mutagenesis enable efficient mapping-by-sequencing. In contrast, its applicability to gamma irradiation-derived mutants has remained largely unexplored because irradiation frequently produces large deletions and complex structural variants that are not optimally captured by short-read Illumina sequencing and may substantially reduce informative polymorphisms required for high resolution mapping.

To address this knowledge gap, we evaluated whether MutMap could successfully identify the causal mutation in a gamma irradiation-derived soybean mutant developed from the elite Vietnamese cultivar DT26. In addition, we assessed the breeding utility of the resulting mutant through functional marker development and introgression into elite genetic backgrounds.

## 2. Materials and Methods

### 2.1. Creation and Characterization of Mutant Soybean Line DT26BS and Mapping Population

Mutation method: ^60^Co gamma irradiation of 64.8 kCi power source at the dose of 150 Gy on 500 dry seed of soybean variety DT26, and an irradiation time of 30 min were used. M1 was grown and harvested in bulk. M2 plants with black seeds were identified, propagated to M6 by single-seed descendant and named DT26BS.

F_1_ seeds obtained from a single DT26BS female crossed with several wild-type DT26 male plants were grown and self-pollinated to generate F_2_ mapping populations. F_2_ seeds were collected separately from individual selfed F_1_ plants, and F_2_ individuals were phenotyped to determine the segregation ratio.

Nutrition content measurement was carried out by the Viet Nam national center for food analysis and assessment (NACEFA-FIRI) according to their standard protocol TCVN as follows: anthocyanin was measured according to FIRI.M.237 using the pH differential method as in [23]; protein content was measured according to TCVN 8125:2015 using the Kjeldahl method [24]; and lipid content was measured according to TCVN 6555:2017 using the Randall method [25].

### 2.2. DNA Samples

The following DNA samples were prepared for whole-genome sequencing:

B1 (wild-type parent): Genomic DNA was extracted from a pooled sample of young leaf tissues collected from 30 DT26 individuals with the wild-type buff seed coat.

F2V (wild-type F_2_ bulk): Genomic DNA was extracted from an equal-mass pooled sample of leaf tissues from 166 F_2_ individuals exhibiting the wild-type buff seed-coat phenotype. All individuals were derived from a single self-pollinated F_1_ plant.

F2D (mutant F_2_ bulk): Genomic DNA was extracted from an equal-mass pooled sample of leaf tissues from 57 F_2_ individuals displaying the mutant black seed-coat phenotype. These individuals were derived from the same F_1_ plant used to generate the F2V population.

Genomic DNA was extracted using the CTAB method [26] followed by clean-up using the QIAGEN DNease Plant Mini kit (Qiagen, Germantown, MD, USA).

### 2.3. Sequencing and Data Processing, MutMap

All DNA samples were sequenced by Novogene (NovogeneAIT Genomics, Singapore) using Illumina technology at an average coverage of 30×. Raw sequencing reads were first assessed for quality using FastQC software (v0.12.1) [27], followed by trimming to remove low-quality bases and short reads using Trimmomatic v0.41 [28]. MutMap analysis was performed using the MutMap pipeline developed by Yu Sugihara [19] (https://github.com/YuSugihara/MutMap; accessed on 15 January 2024).

### 2.4. PCR Analysis

PCR was performed following standard procedures. Amplification reactions were established using DreamTaq (Thermo Fisher Scientific, Waltham, MA, USA) in a total volume of 20 µL, including 2 µL of Master Mix 10×, 0.5 µL of each of the forward and reverse primers (10 µM), and 100 ng of genomic DNA. Amplification was done in Eppendorf Mastercycler Nexus X2 with the following thermal conditions: (1) predenaturation at 95 °C for 2 min; (2) denaturation at 95 °C for 30 s; (3) annealing at 57 °C for Del12bp or 55 °C for the two primers of Fragment 3 and 9, for 30 s; and (4) extension at 72 °C for 2 min, repeated from step 2 to step 4 for 35 cycles, and followed by a final extension at 72 °C for 10 min. Amplification products were analyzed by electrophoresis in agarose gel containing stain solution Redsafe^TM^ and photographed by UV Infinity Vilber Lourmat Imaging Systems (Vilber Lourmat, Collégien, France).

All primers used are listed in Table S1.

## 3. Results

### 3.1. Isolation and Characterization of the DT26BS Mutant

DT26 is a Vietnamese commercial soybean variety released in 2008, derived from a cross between DT2000 and DT12. To generate novel genetic variation for mutation breeding, dry seeds of DT26 were exposed to gamma irradiation using a ^60^Co source with an activity of 64.8 kCi. For each treatment, 500 seeds were irradiated at doses ranging from 100 to 350 Gy for 30 min. The effects of irradiation and the frequency of induced mutations are summarized in Table S2. Among the tested treatments, irradiation doses between 150 and 250 Gy provided the most effective balance between mutation induction and plant survival.

**Figure 1:**
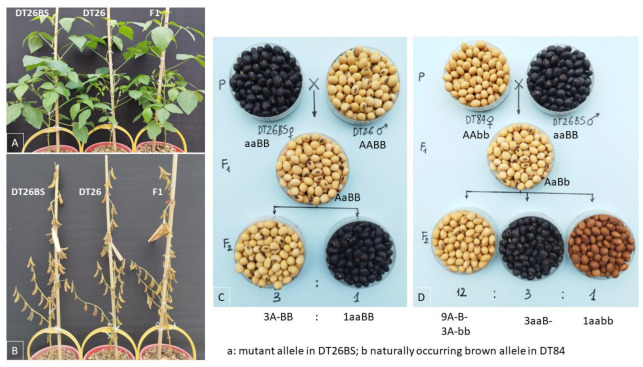
Phenotype of wild-type DT26 and black seed mutant DT26BS. Whole plant phenotype at flowering stage (**A**) and harvesting stage (**B**) of DT26BS (DT26 Black Seed mutant); DT26 (wild-type); and F_1_ (F_1_ plant from cross between DT26BS (used as female) and DT26 (used as male)). Self-pollinated seed of the parents, F_1_ plants and F_2_ plants from cross DT26BS × DT26 (**C**) and from cross DT84 × DT26BS (**D**) with illustrated phenotypic and genotypic segregation ratio.

In the M_2_ generation derived from the 150 Gy treatment, a mutant with a black seed coat was identified, in contrast to the buff seed coat of the wild-type DT26. After six generations of selfing (M_1_–M_6_), the line showed complete homozygosity and stable inheritance of the black seed-coat phenotype. This mutant line was designated DT26BS (DT26 BlackSeed) (Figure 1).

DT26BS exhibited bio-agronomic characteristics comparable to those of the wild-type DT26, including plant architecture, growth duration, and yield (Figure 1A,B; Table S3). The two lines differed mainly in seed-coat color, hilum color, and anthocyanin content. While anthocyanin was not detected in the wild-type DT26, DT26BS accumulated 42.45 ± 1.83 mg/100 g anthocyanin in the seed. However, this level is lower than that reported in many Korean and Chinese black soybean landraces, which can contain between approximately 98 and over 2000 mg/100 g of anthocyanin [1,5].

Importantly, the total protein and lipid contents of DT26BS were similar to those of DT26, indicating that the mutation primarily affects seed pigmentation without major impacts on nutritional composition (Table S3).

### 3.2. Genetic Inheritance of the Black Seed-Coat Phenotype

To investigate the genetic basis of the black seed-coat phenotype, DT26BS plants were crossed with the wild-type DT26. DT26BS was used as the female parent and DT26 as the male parent. The parental lines and the resulting F_1_ plants displayed indistinguishable vegetative and reproductive phenotypes, including plant architecture and pod morphology (Figure 1A,B), confirming that DT26BS shares the same genetic background as DT26 and did not arise from contamination with another variety.

Because the seed coat is derived from maternal tissue, its color is determined by the genotype of the maternal plant. All seeds from selfed F_1_ plants were buff (Figure 1C), and the F_2_ population segregated in a ratio of 166 buff to 57 black (3:1; chi-square test, *p* = 0.88), indicating that the black seed-coat phenotype is controlled by a single recessive mutation.

Interestingly, while making crosses between DT26BS and two unrelated soybean varieties (DT84 and DT2010) to generate mapping population, an additional brown seed-coat phenotype alongside black was observed, suggesting interaction with other loci. In the DT84 × DT26BS cross, the F_2_ population segregated in a 12 buff:3 black:1 brown ratio (93:22:7; chi-square test, *p* = 0.91) (Figure 1D), supporting dominant epistasis involving two loci. Under this model, a dominant allele at the first locus (*A*) masks the effect of the second locus (*B*), producing buff seeds. In the recessive *aa* background, the *B* locus determines pigmentation, with *aaB*-producing black seeds and *aabb* producing brown seeds. A similar segregation pattern was observed in the DT26BS × DT2010 cross (Figure S1).

Together, these results indicate that the black seed-coat phenotype in the DT26BS background is controlled by a single recessive mutation that can interact with a second locus in other genetic backgrounds through dominant epistasis to generate a brown seed-coat phenotype.

### 3.3. MutMap Analysis Identifies a Candidate Genomic Region

**Figure 2:**
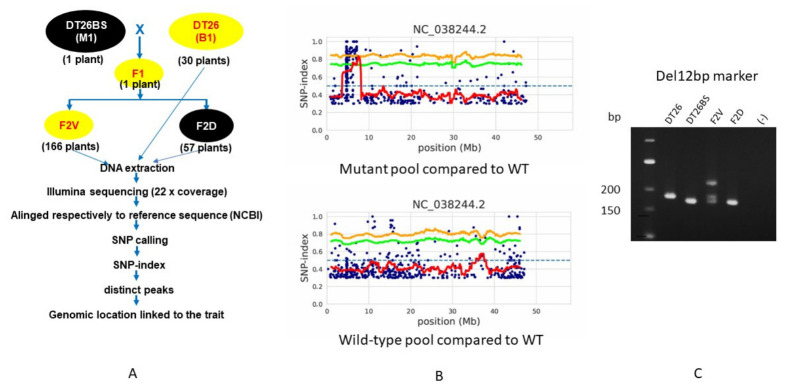
MutMap results. (**A**) MutMap pipeline for mapping DT26BS; F2V: pool of F_2_ individuals with buff seeds; F2D: pool of F_2_ individuals with black seeds; B1: pool of DT26 wild-type plants; M1: one individual DT26BS mutant (used as female in the cross). The color of the seed coat of the plants (black or buff) was symbolized with an oval filled with either black or yellow color. (**B**) SNP index of the peak found on chromosome 8. Mean SNP index (proportion of reads containing the alternative allele relative to wild-type at each genomic position) plot along contig NC_038244.2 (chromosome 8) of F_2_ mutant pool vs. wild-type and F_2_ wild-type pool vs. wild-type; only SNP ratios over 0.3 (so true SNP) were plotted. Blue dot: variant; red line: mean SNP index; green line: mean p95; orange line: mean p99. p95: 95% confidence interval of simulated SNP index; p99: 99% confidence interval of simulated SNP index. (**C**) PCR analysis showed linkage of Del12bp marker to the black seed trait. (-): negative control using water as template.

To identify the genetic lesion responsible for the black seed-coat phenotype, we applied the MutMap approach [18]. Three DNA samples were sequenced: the wild-type parent (B1), a bulk of 57 F_2_ individuals with black seeds (F2D), and a bulk of 166 F_2_ individuals with buff seeds (F2V) (Figure 2A). All F_2_ individuals were derived from a single F_1_ plant to ensure uniform genetic background for MutMap analysis.

Whole-genome sequencing using the Illumina HiSeq platform generated 52–71 Gb of data per sample, corresponding to an average genome coverage of 21–24× for the soybean genome (978.4 Mb) (Table 1). Sequencing reads were aligned to the *G.max* reference genome (Williams 82, version 4.0). Approximately 1.6 million sequence variants were detected relative to the reference genome in each sample.

Following the MutMap bioinformatic pipeline [19], the SNP index, defined as the proportion of reads containing the alternative allele relative to wild-type at each genomic position, was calculated across the genome. Two SNP-index plots were generated: one comparing the mutant F_2_ bulk (F2D) with the wild-type parent (B1), and another comparing the wild-type F_2_ bulk (F2V) with B1 (Figure 2B and Figure S2).

A clear SNP-index peak was detected exclusively in the F2D vs. B1 comparison, but not in F2V vs. B1, indicating linkage to the causal mutation. SNPs within this peak exceeded the statistical significance threshold, and several reached an SNP-index value of 1, suggesting tight linkage with the causal mutation. These candidate variants were localized to a genomic region spanning 4–10 Mb on chromosome 8.

### 3.4. Validation of the MutMap Candidate Region

To validate the candidate region identified by MutMap, PCR markers based on insertion–deletion polymorphisms were developed. A 12 bp deletion (Del12bp) located within the mapped region (7.119 Mb on chromosome 8) was selected for genotyping (Figure 2C and Figure S3).

Genotyping of the DT26 × DT26BS F_2_ population revealed strong linkage between the Del12bp marker and the black seed phenotype. Among the 57 black seed individuals, only seven were heterozygous at this marker, whereas only three of the 166 buff seed individuals carried the mutant allele in homozygous form (Figure S4). These results correspond to an estimated genetic distance of 4.48 cM between the marker and the causal mutation. A similar analysis was performed in a second mapping population, the DT84 × DT26BS F_2_ population, where four recombinants were detected among 122 individuals, corresponding to a genetic distance of 3.3 cM (Figure S5). These results from two independent mapping populations confirm that the causal mutation lies in close proximity of 3.3 to 4.5 cM to the Del12bp marker on chromosome 8.

### 3.5. Identification of Structural Variation in the CHS Locus

Considering that irradiation mutagenesis can generate structural-variant mutations, we examined the candidate genomic interval for deletions using read alignment visualization in Integrative Genomics Viewer (IGV version 2.11.0) [11]. Several deletions were detected in the mutant that were absent in the wild-type parent.

**Figure 3:**
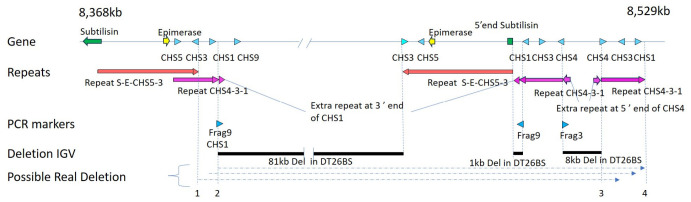
Diagram of the 158.8 kb of *CHS* clusters from 8368 kb to 8529 kb on chromosome 8. Gene: *CHS* homologs are shown in a cyan triangle arrow, *epimerase* in yellow, and *subtilisin* in green. Note that 5′ end *subtilisin* is truncated. Only genes related to *CHS* homologs and in the repeat regions are shown. Repeats: position of different types of repeating unit present in *CHS* clusters. PCR markers: position of PCR marker Frag9 and Frag3. Deletion IGV: deletions found in MutMap as suggested by alignment of read from IGV program. Possible Real Deletion (dash lines): deduced possible real deletion due to misalignment of repetitive regions: deletion may start anywhere between point 1 and 2; ending at least passes point 3, extending furthest up to point 4.

Three apparent deletions were observed: an ~81 kb deletion, a ~1 kb deletion, and an ~8 kb deletion within the candidate region (Figure 3 and Figure S6A,B). Notably, this genomic region corresponds to the well-known *I* locus [11], which contains clustered *Chalcone Synthase* (*CHS*) genes arranged in inverted repeats. These repeats produce endogenous RNA interference signals that silence other *CHS* genes in the seed coat, thereby preventing anthocyanin accumulation and resulting in the buff seed color.

Closer examination of the local repeat structure suggested that these apparent deletions likely represent a single large continuous deletion of approximately 176 kb, rather than three independent deletions. The intervals between the apparent deletions correspond precisely to known *CHS* repeat units (Figure 3), suggesting that reads flanking each repeat were misaligned due to the highly repetitive nature of the locus. Consistent with this interpretation, reads within these intervals appear white/transparent in IGV (Figure S6A,B), indicative of ambiguous or repetitive alignments caused by missing or improperly aligned mate pairs. We therefore propose that DT26BS harbors a single large continuous deletion of at least ~176 kb, spanning multiple *CHS* repeat units within the I locus.

### 3.6. Marker Validation of CHS Deletions

To experimentally validate deletions within the *CHS* locus, PCR markers were designed targeting regions spanning *CHS* genes and adjacent border regions. Due to the high sequence similarity among *CHS* homologs, markers were designed based on regions previously characterized in [11].

**Figure 4:**
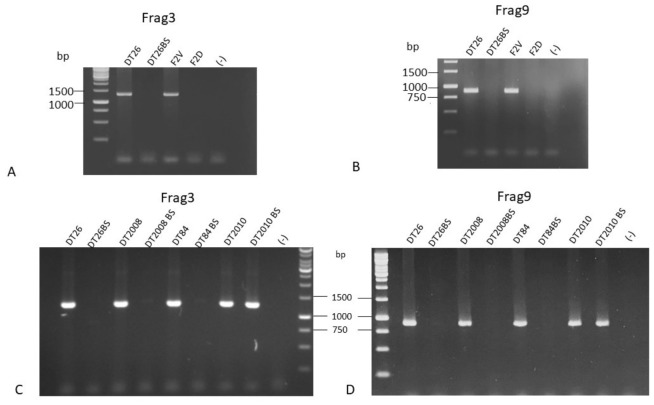
PCR reveals deletion in *CHS* locus in DT26BS and other black seed varieties. (**A**) DNA samples used for MutMap (Frag3 marker); (**B**) DNA samples used for MutMap (Frag9 marker); (**C**). DNA from different varieties (Frag3 marker); (**D**) DNA from different varieties (Frag9 marker). BS: black seed. (-) water.

Two markers corresponding to Fragment 3 (Frag3) and Fragment 9 (Frag9) were tested (Figure 3 and Figure 4). Both fragments were present in wild-type plants but absent in the mutant bulk (F2D), indicating deletion of these regions in DT26BS (Figure 4A,B). Frag9, which produced a smaller and more robust PCR amplicon, was selected for further analysis.

Genotyping of the F_2_ mapping population, using Frag9 confirmed complete linkage between the Frag9 deletion marker and the black seed phenotype in the DT26 × DT26BS population (comprising 223 individuals) and also linkage to both the black and brown seed phenotype in the DT84 × DT26BS population (comprising 122 individuals) (Figures S7 and S8).

### 3.7. Independent Soybean Black Seed Mutants Support Involvement of the CHS Locus

To further examine whether *CHS* deletions are a common cause of black seed mutants, we analyzed three additional black seed mutants identified independently from irradiated–mutagenized populations of the soybean varieties DT2008, DT84, and DT2010.

PCR genotyping revealed that the DT2008-Black seed and DT84-Black seed mutants also lacked both the Frag9 and Frag3 markers, suggesting *CHS* clusters were also not intact in these mutants (Figure 4C,D). In contrast, the DT2010-Black seed mutant showed a wild-type Frag3 and Frag9 genotype, indicating that the black seed phenotype in DT2010-Black seed may result from a different genetic lesion. Further genetic tests will be required to determine whether this mutation in DT2010-Black seed affects a different gene or represents a distinct alteration within the *CHS* locus.

### 3.8. Development of New Black Soybean Breeding Lines

To develop black soybean varieties with shorter growth duration and improved yield, DT26BS was crossed with two early-maturing varieties, DT84 and DT2010, in spring 2022. Using the pedigree selection method from the F_2_ to F_7_ generations, 17 superior lines were selected from three cross-combinations. Preliminary evaluation of the F_7_ generation indicated that these lines exhibited growth durations of 78–85 days and yields of 2.07–2.55 tons per hectare (Table S4, Figure S9), meeting the breeding targets for early maturity and high productivity. These results indicate that the DT26BS mutation is stable and transferable and does not appear to be linked to undesirable agronomic traits, making it a useful genetic resource for breeding black seed soybean varieties.

## 4. Discussion

### 4.1. Utility of Gamma-Irradiation and MutMap in Soybean Mutant Breeding

Gamma-ray irradiation has been used to induce genetic variation in soybean. Feng et al., 2023 [16] reported an optimal dose range of 263–343 Gy (source: ^137^Cs) for the cultivar Williams 82; our results showed that useful mutations can also be obtained at lower irradiation doses. For Vietnamese varieties, such as DT26, we found that 150–250 Gy (source: ^60^Co) gamma rays was the best range for balancing mutation frequency and minimizing detrimental effects on other agronomic traits. These observations suggest that the optimal irradiation dose is genotype-dependent, and should be determined according to the breeding objectives and desired mutation spectrum.

Previous applications of MutMap have primarily focused on identifying point mutations, often derived from EMS mutagenesis only [18,20,21,22], and to our knowledge, reports applying MutMap to gamma irradiation-derived mutant populations remain extremely limited. The present study demonstrates the effectiveness of MutMap for mapping a gamma-irradiated mutant. Although irradiation can induce large deletions that may be difficult to detect using short-read sequencing, our results show that sufficient polymorphic markers remain available for effective mapping. This is in agreement with [16] where they found many single-base substitution and small indels induced by gamma rays.

The identification of a ~176 kb deletion spanning the *CHS* gene cluster highlights the capability of this approach to resolve complex genomic lesions beyond single-nucleotide changes. Importantly, our analysis was conducted on an elite tropical soybean cultivar with a genetic background distinct from standard reference genomes, yet it still enabled accurate localization of the causal region. These findings highlight the applicability of MutMap to structurally complex mutations and non-reference germplasm, supporting its broader use in crop improvement programs, particularly in breeding-relevant genetic backgrounds.

With an average sequencing depth of ~20×, and only three samples needed for sequencing (wild-type, F_2_ mutant pool and F_2_ wild-type pool), a small F_2_ population (in our case 57 mutant and 166 wild-type F_2_ individuals), even though the genetic background (DT26) is unrelated to the reference genome Williams 82, this approach provided a cost-effective, rapid, and practical means of localizing the causal mutation to a defined genomic interval.

However, the mapping resolution was limited (in our case 3–5 cM), likely due to the relatively small population size and moderate sequencing depth. Consequently, additional analyses such as PCR marker validation were required to identify the structural mutation responsible for the phenotype.

### 4.2. Disruption of the CHS Inverted Repeat at the I Locus of DT26BS Mutant

Our genetic and molecular analyses strongly suggest that the black seed phenotype in DT26BS is caused by deletion of repeat units within the *CHS* cluster at the I locus on chromosome 8. Previous studies have shown that inverted repeats of *CHS* genes at this locus produce double-stranded RNA that triggers RNA interference (RNAi), silencing *CHS* expression specifically in the seed coat [29]. This silencing prevents anthocyanin biosynthesis and results in a dominant buff seed phenotype.

The large deletion identified in DT26BS disrupts this inverted-repeat structure, likely abolishing RNAi-mediated silencing and allowing *CHS* expression to resume in the seed coat. Restoration of *CHS* activity would permit flavonoid and anthocyanin accumulation, thereby producing the black seed phenotype observed in DT26BS.

### 4.3. Organization of the I Locus in Soybean: The CHS Cluster

Because the *CHS* locus is structurally complex, we revisited previous studies to integrate current knowledge of its organization.

Based on BAC analysis and sequencing, Tuteja and Vodkin [30] proposed that the soybean *CHS* locus is a complex region spanning more than 230 kb. Using the *G. max* reference genome assembly v4.0, we confirmed this finding and further resolved the organization of the *CHS* repeat clusters (Figure 3).

Three *CHS* clusters were identified. The first comprises *CHS5*-*CHS3*-*CHS1*-*CHS9*, followed ~81 kb downstream by a second cluster (*CHS3*-*CHS5*). The third cluster consists of six *CHS* genes arranged as a perfect inverted repeat (*CHS1*-*CHS3*-*CHS4*-*spacer*-*CHS4*-*CHS3*-*CHS1*). These clusters arose from two principal repeating blocks. The first is a 21.3 kb unit (Rep *S*-*E*-*CHS5*-*CHS3*), repeated two times in inverted directions, 91 kb apart. The second is a 9.7 kb core repeat (*CHS4*-*CHS3*-*CHS1*), present in three copies with minor variation at their 5′ and 3′ extensions (Figure 3). Consistent with previous reports [11,14,30], we identified a ~10.9 kb perfect inverted repeat at the I locus, comprising a 9761 bp *CHS4-3-1* core and an ~1.1 kb 5′ upstream extension of *CHS4*.

Notably, at least one copy of the *CHS5*-*CHS3*-*CHS1*-*CHS9* repeat remains intact in DT26BS and includes a tail-to-tail (inverted) arrangement of *CHS5* and *CHS3*. These results suggest that the *CHS5-CHS3* tail-to-tail arrangement configuration alone appears insufficient to generate the effective double-stranded RNA required to trigger endogenous silencing. This suggests that the inhibitory *I* locus responsible for the buff seed phenotype requires the longer inverted repeats *CHS4*-*CHS3*-*CHS1* and/or the Rep *S-E-CHS5-CHS3* repeats.

The Frag9 PCR results from independent black seed-coat mutants were unexpected and warrant further consideration. Frag9 occurs as an inverted repeat at two loci ~104 kb apart; thus, loss of amplification could result from either two separate deletions or a single large deletion spanning both copies. Notably, three independent mutants lacked Frag9, suggesting recurrent irradiation-induced structural variation at this locus, potentially due to the repetitive architecture of the *CHS* cluster predisposing it to irradiation-induced deletions.

### 4.4. Future Study

The genetic basis of the brown seed-coat phenotype observed in the DT84 × DT26BS population remains unresolved and warrants further investigation. A MutMap approach using a brown-seeded bulk from this population would enable rapid mapping of the underlying locus. Given that brown pigmentation likely reflects partial disruption of the flavonoid pathway, candidate genes may include regulators or enzymes acting downstream or parallel to *CHS*, such as *dihydroflavonol 4-reductase* (*DFR*), *anthocyanidin synthase* (*ANS*), or transcription factors controlling flavonoid biosynthesis [31,32,33]. Identifying this locus would provide insight into genetic interactions modulating seed-coat pigmentation in soybean.

Future studies should investigate whether the irradiation-induced deletion of the CHS gene cluster influences the accumulation of other flavonoid-derived metabolites, particularly isoflavonoids, to better understand the broader metabolic consequences of this structural variant and its implications for soybean nutritional quality.

### 4.5. Implications for Soybean Breeding

The DT26BS mutant provides a useful genetic resource for developing improved black soybean varieties. Importantly, the mutation does not negatively affect major agronomic traits such as yield or protein content. Anthocyanins present in black soybean seeds have been widely reported to possess beneficial health effects, including anti-inflammatory, anti-obesity, and antidiabetic activities [7,8]. Preliminary breeding efforts have already produced several promising early-maturing black soybean lines with growth durations below 90 days and yields exceeding 2.0 tons per hectare.

These results demonstrate that mutation breeding combined with modern genomic analysis can efficiently generate novel germplasm for crop improvement.

## 5. Conclusions

In conclusion, we developed a gamma-irradiated mutant population in the elite Vietnamese soybean cultivar DT26 and identified a stable black seed-coat mutant, DT26BS, with no adverse effects on major agronomic or nutritional traits. Using a MutMap-based mapping-by-sequencing strategy, we successfully localized the causal mutation to a region on chromosome 8 and demonstrated that the phenotype is associated with a large (~176 kb) structural deletion spanning the *Chalcone Synthase* (*CHS*) gene cluster at the *I* locus. As suggested by other studies on the *CHS* locus, this deletion likely disrupts the inverted-repeat architecture responsible for RNAi-mediated *CHS* silencing, leading to restoration of anthocyanin accumulation and the black seed-coat phenotype. Our results further confirm the utility of MutMap for rapidly identifying complex irradiation-induced structural variants in non-reference, elite tropical germplasm. The gamma irradiation–MutMap pipeline presented here provides an efficient framework for forward genetics in soybean and supports the development of improved black soybean breeding lines with enhanced nutritional value and agronomic performance. More broadly, our findings support the wider application of this MutMap pipeline in other crop species where irradiation mutagenesis and breeding is routinely employed.

## Figures and Tables

**Table 1:** Sequencing data summary.

Sample	Wild-Type B1	F_2_ Mutant Pool F2D	F_2_ Wild-Type Pool F2V
Number of mapped reads	159,350,290	145,297,272	152,222,526
Mean coverage	23.8	21.7	22.7
Total number of variants *	1,460,775	1,687,851	1,667,429
Number of SNPs *	1,158,776	1,319,932	1,326,205
Number of insertions *	152,160	189,374	189,153
Number of deletions *	149,839	178,545	179,245

*: Variants of the sample compared to William82 reference genome.

## Data Availability

The sequencing data are available in the NCBI database (https://www.ncbi.nlm.nih.gov (accessed on 13 July 2026)) under the SRA accession numbers SRR37455607, SRR37252327 and SRR37252326 for the B1, F2V and F2D samples, respectively.

## Supplementary Materials

The following supporting information can be downloaded at: https://www.mdpi.com/article/10.3390/genes17070814/s1, Figure S1. Segregation of seed coat color in F_2_ population of DT26BS/DT2010. Figure S2. Mutmap SNP-index plot across entire genome containing 20 chromosomes of soybean. Figure S3. IGV view showing a deletion 12 bp from position 7.119.700-7.119.713. Figure S4. PCR genotyping of the F_2_ population from DT26BS × DT26 using the Del12bp marker. Figure S5. PCR genotyping of the F_2_ population from DT84 × DT26BS-12 using the Del12bp marker. Figure S6. IGV view of deletion within Mutmap peak. Figure S7. PCR genotyping of the F_2_ population from DT26BS × DT26 using the Frag9 marker. Figure S8. PCR genotyping of the F_2_ population from DT84 × DT26BS-12 using the Frag9 marker. Figure S9. Development of new black soybean breeding lines in F7 generation. Table S1: Sequences of Primers used for validation of 12-bp deletion and regions locating CHS locus. Table S2. Effect of different doses of radiation on survival and phenotypic mutant frequency. Table S3. Bio-agronomic characteristics and some nutrition content of DT26 and DT26BS. Table S4. Some characters of F7 lines with black seed coat derived from the combination DT84/DT26BS, DT26BS/DT84 and DT2010/DT26BS in Dan Phuong—Hanoi in winter 2025.
