# Supplementary material for: MutMap Reveals a Structural Deletion at the Chalcone Synthase Locus Controlling Black Seed Coat in a Gamma-Irradiated Vietnamese Soybean Mutant
Source: Genes (Basel). 2026 Jul 17;17(7):814. doi: 10.3390/genes17070814 (PMC13409758; doi:10.3390/genes17070814)
Supplement: Supplementary file 1 [file genes-17-00814-s001.zip › Supplemental Figures v3.pdf]

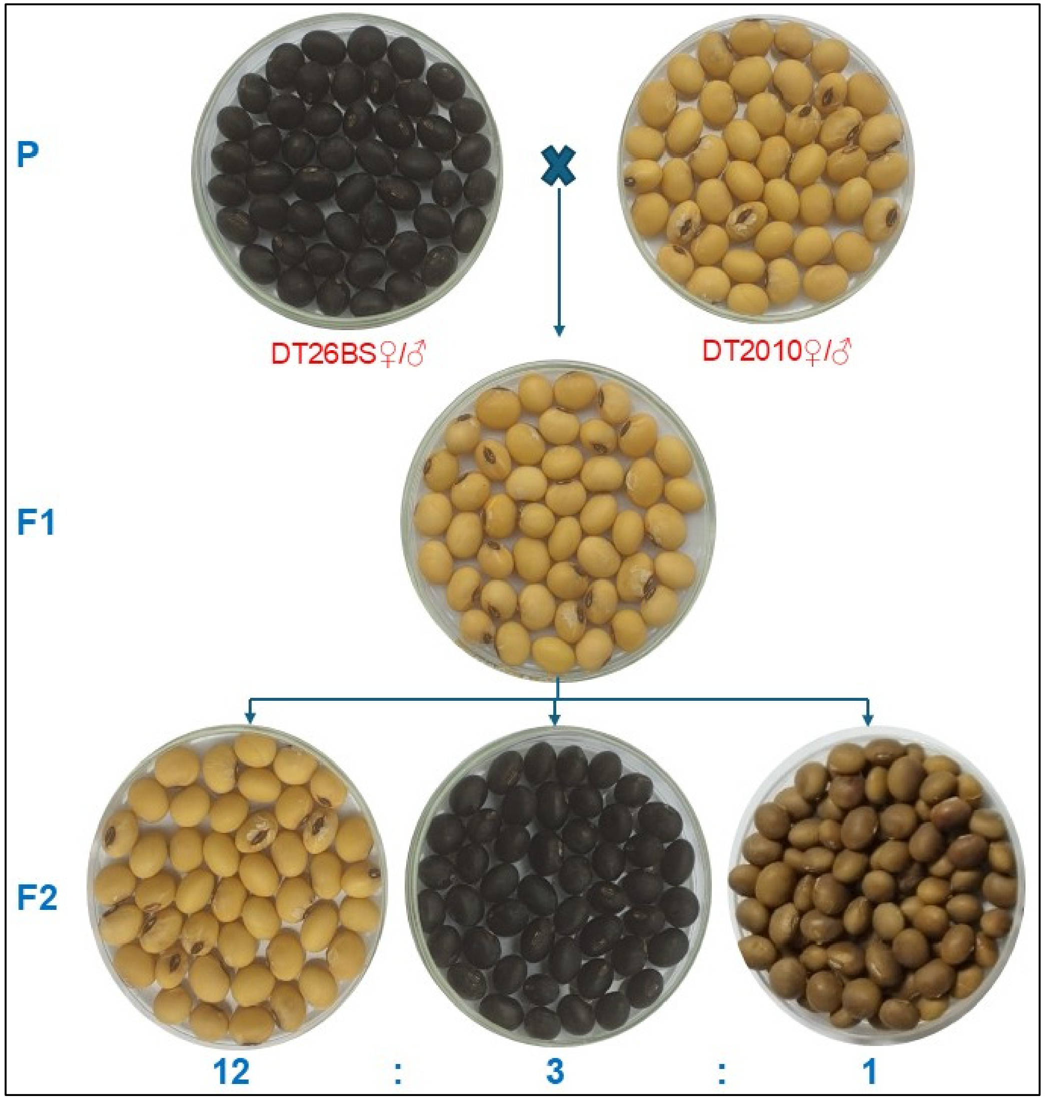

Figure S1. Segregation of seed coat color in F2 population of DT26BS/DT2010

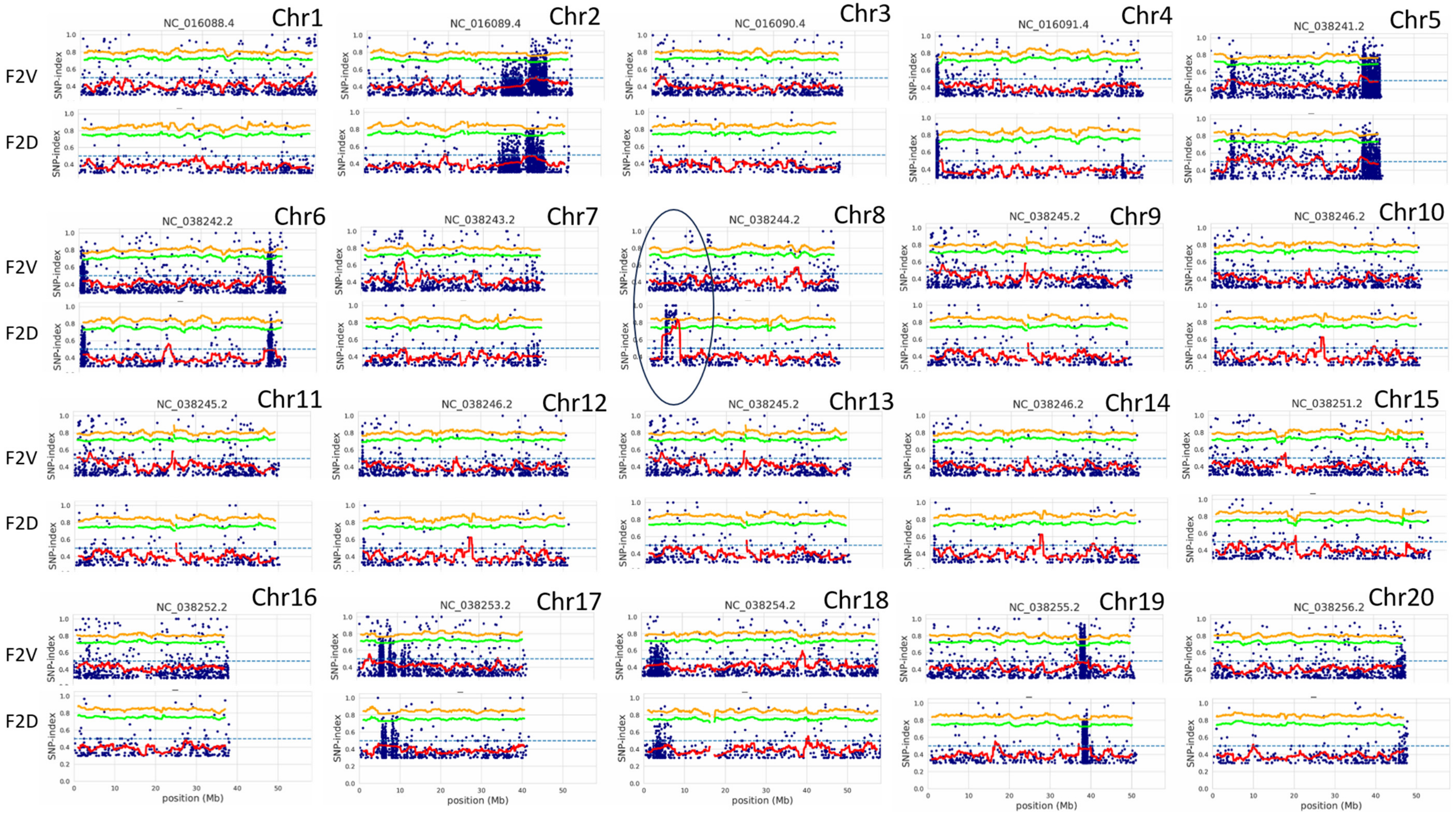

Figure S2. Mutmap SNP-index plot across entire genome containing 20 chromosomes of soybean.

Compare the figures from F2 mutant pool (F2D) vs wild-type (B1) and F2 wild-type pool (F2V) vs wild-type (B1); only SNP ratio over 0.3 (so true SNP) were plotted. Blue dot: variant; Red line: mean SNP-index; Green line: mean P95; orange line: mean p99. p95: 95% confidence interval of simulated SNP-index; p99: 99% confidence interval of simulated SNP-index.

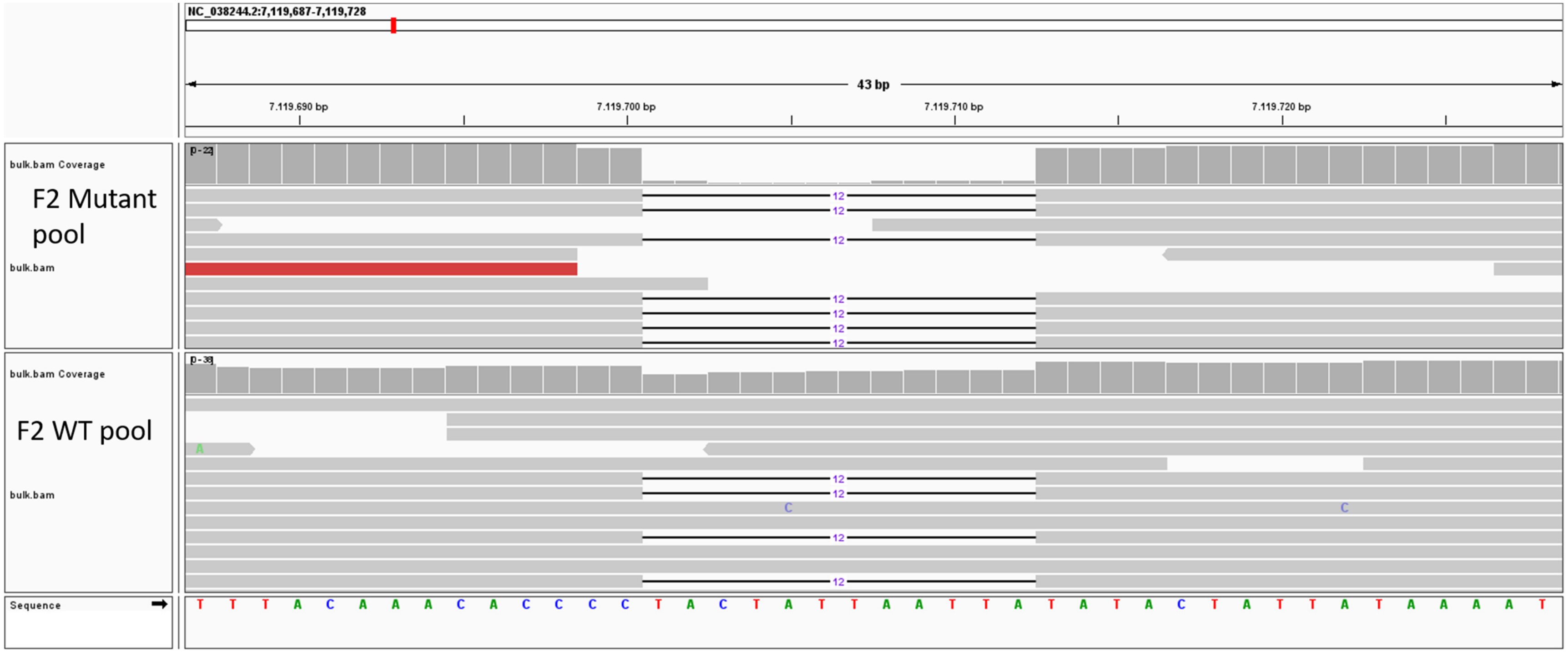

Figure S3. IGV view showing a deletion 12bp from position 7.119.700-7.119.713

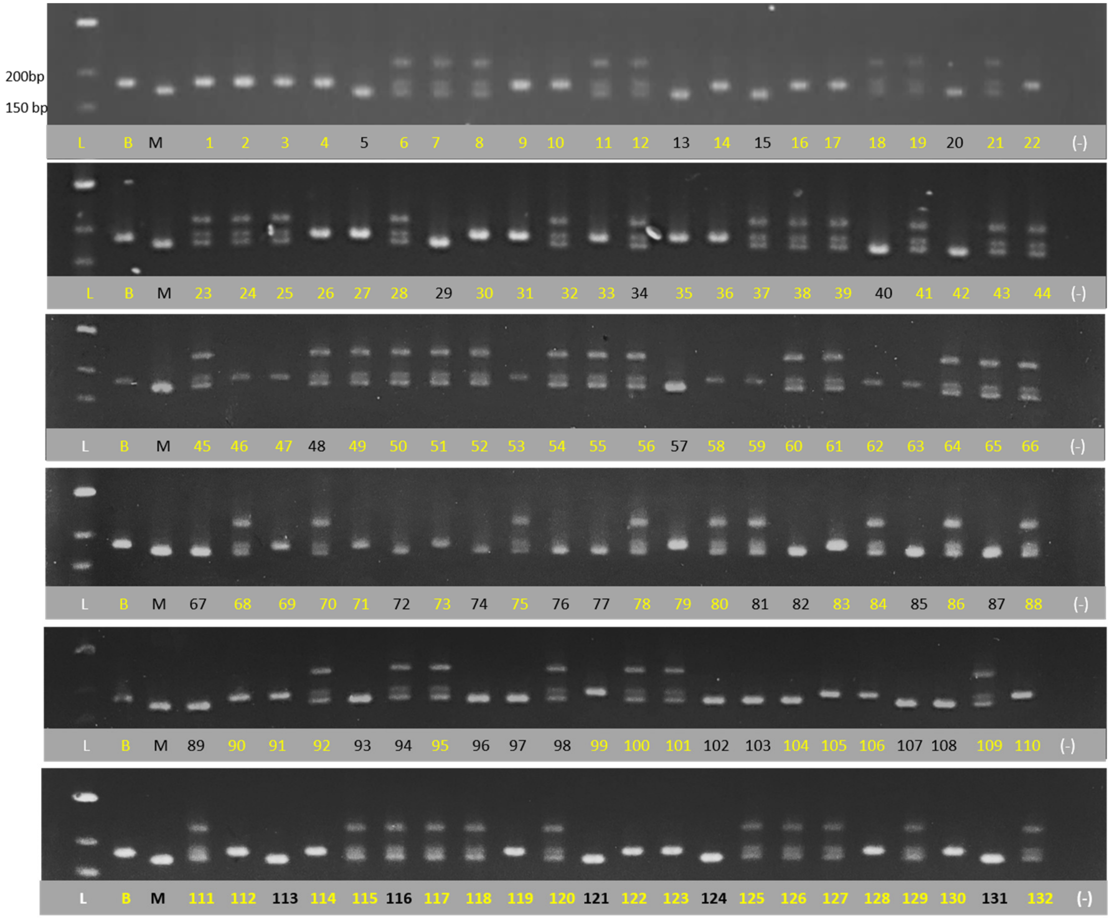

Figure S4. PCR genotyping of the F_2 population from DT26BS × DT26 using the Del12bp marker

Total 223 individuals (numbered 1 to 223) in which 166 have wild-type yellow seeds and 57 have mutant black seeds. L: DNA ladder; B: wild-type; M: mutant; (-) water negative PCR control. Each numbers represents one individual, with font colour to indicate phenotype: yellow font, yellow seeds; black font, black seeds. Recombinant samples were identified among segregating individuals. Numbers in parentheses denote sample IDs: Yellow seeds (42, 104, 195) showed a homozygous mutant band, whereas black seeds (34, 48, 81, 94, 98, 116, 138) showed heterozygous bands.

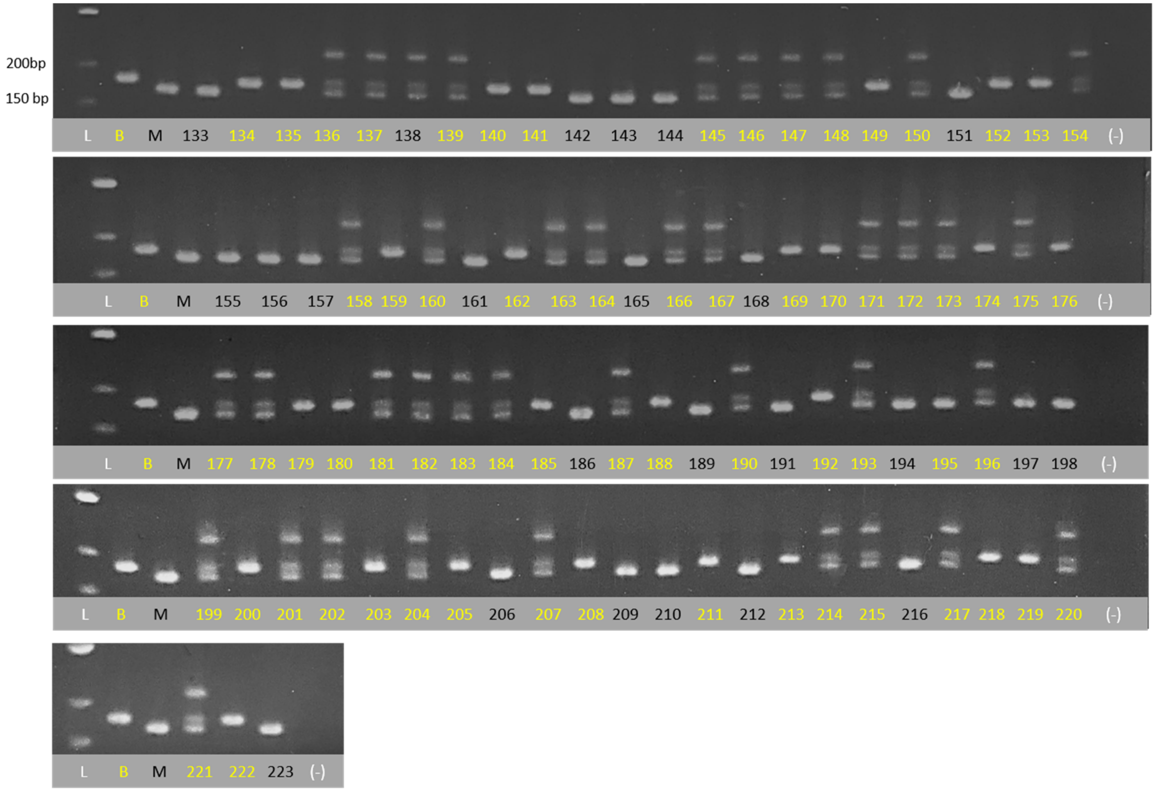

Figure S4. PCR genotyping of the F_2 population from DT26BS × DT26 using the Del12bp marker (cont)

Total 223 individuals (numbered 1 to 223) in which 166 have wild-type yellow seeds and 57 have mutant black seeds. L: DNA ladder; B: wild-type; M: mutant; (-) water negative PCR control. Each numbers represents one individual, with font colour to indicate phenotype: yellow font, yellow seeds; black font, black seeds. Recombinant samples were identified among segregating individuals. Numbers in parentheses denote sample IDs: Yellow seeds (42, 104, 195) showed a homozygous mutant band, whereas black seeds (34, 48, 81, 94, 98, 116, 138) showed heterozygous bands.

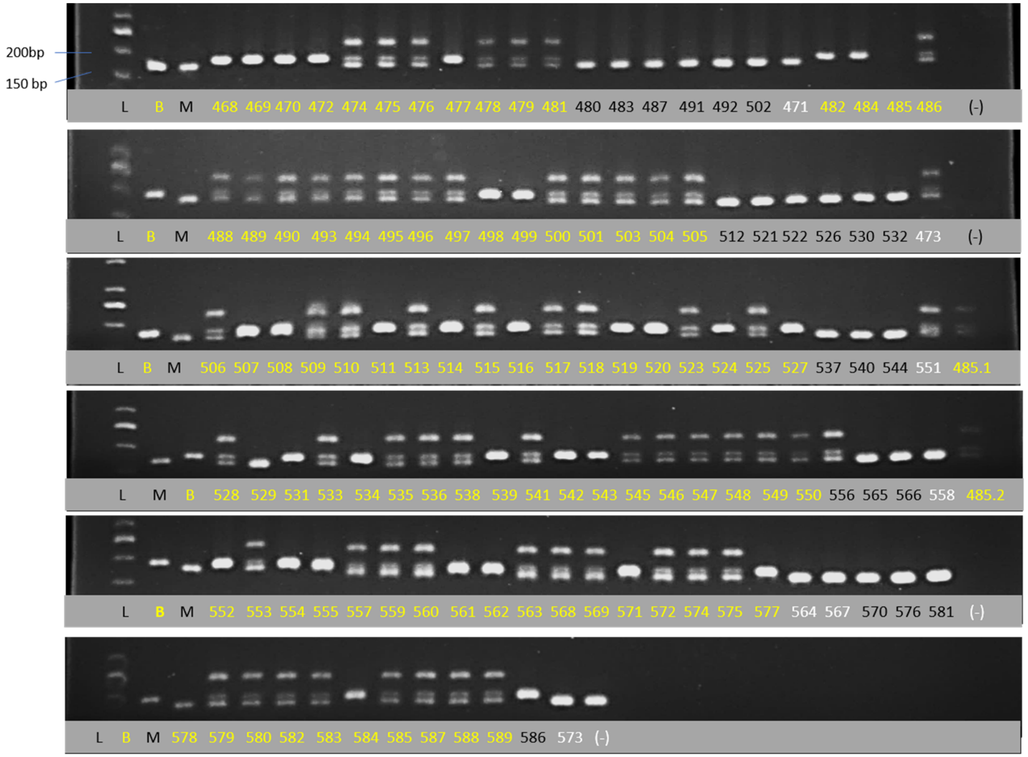

Figure S5. PCR genotyping of the F_2 population from DT84 × DT26BS-12 using the Del12bp marker

A total of 122 individuals (numbered 468–589) were analyzed, including 93 plants with yellow seeds, 22 with black seeds, and 7 with brown seeds. L, DNA ladder; B, wild-type control; M, mutant control; (–), water negative control. Each number represents an individual, with font color indicating phenotype (yellow font, yellow seeds; black font, black seeds; white font, brown seeds).

Recombinant samples were identified among segregating individuals. Numbers in parentheses denote sample IDs. One plant with yellow seed (529) showing a homozygous mutant band, one plant with black seed (556) showing heterozygous bands, and two plants with brown seeds (473 and 551) also showing heterozygous bands.

Note: Sample 485 failed PCR in the initial run and was re-amplified in a subsequent gel (lanes 485.1 and 485.2)

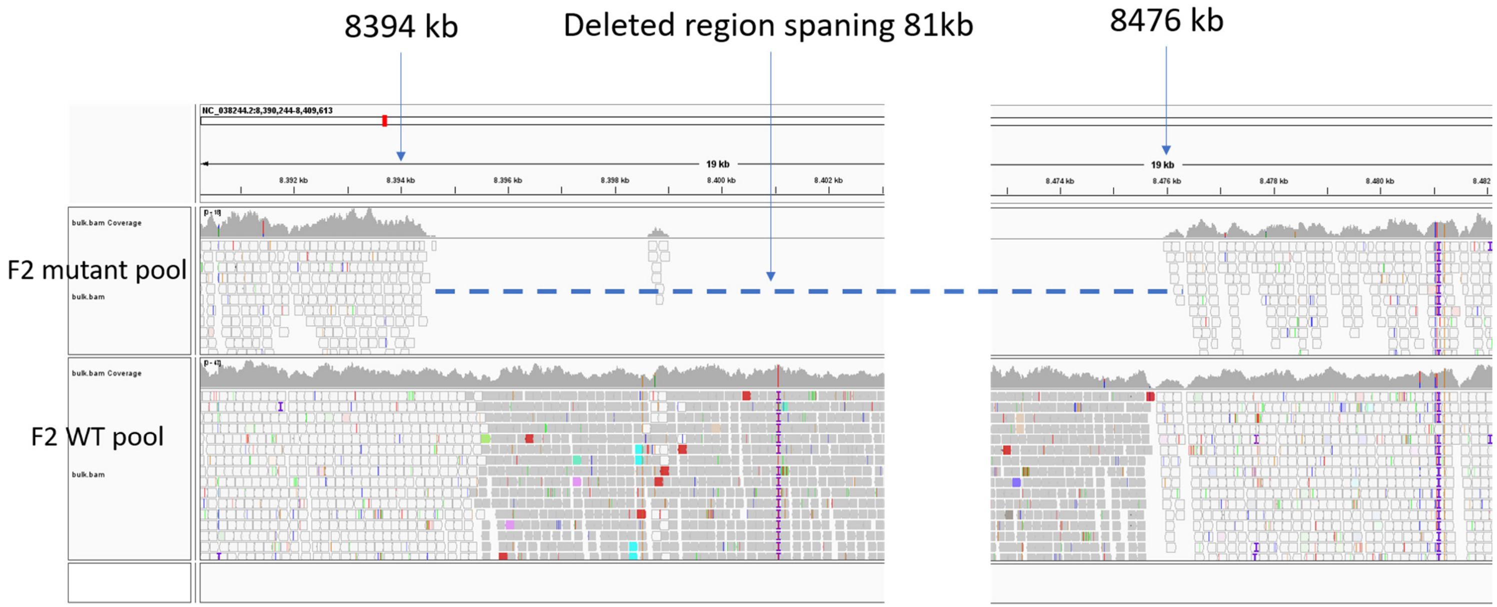

Figure S6. IGV view of deletion within Mutmap peak.

A. IGV view of the 82kb deleted region in mutant pool compared to wild-type, showing the beginning and the end positions of the deletions. Note the mapped read flanking the insertion have the white/transparent color suggesting repetitive region/unmapped mate pair/multiple mapping.

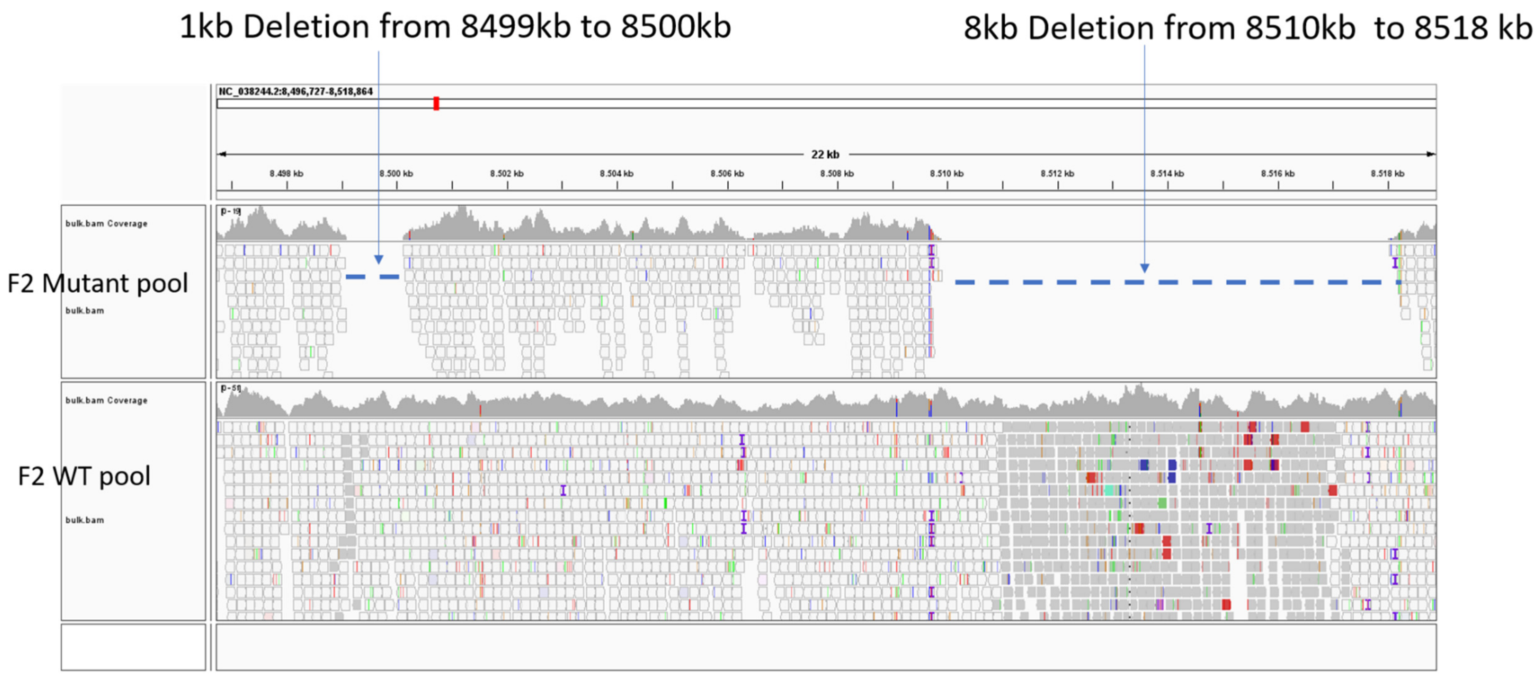

Figure S6 (cont.). IGV view of deletion within Mutmap peak.

B. IGV view of the 1kb and 8kb deleted region in mutant pool compared to wild-type. Note the mapped read flanking the insertion have the white/transparent colour suggesting repetitive region/unmapped mate pair/multiple mapping.

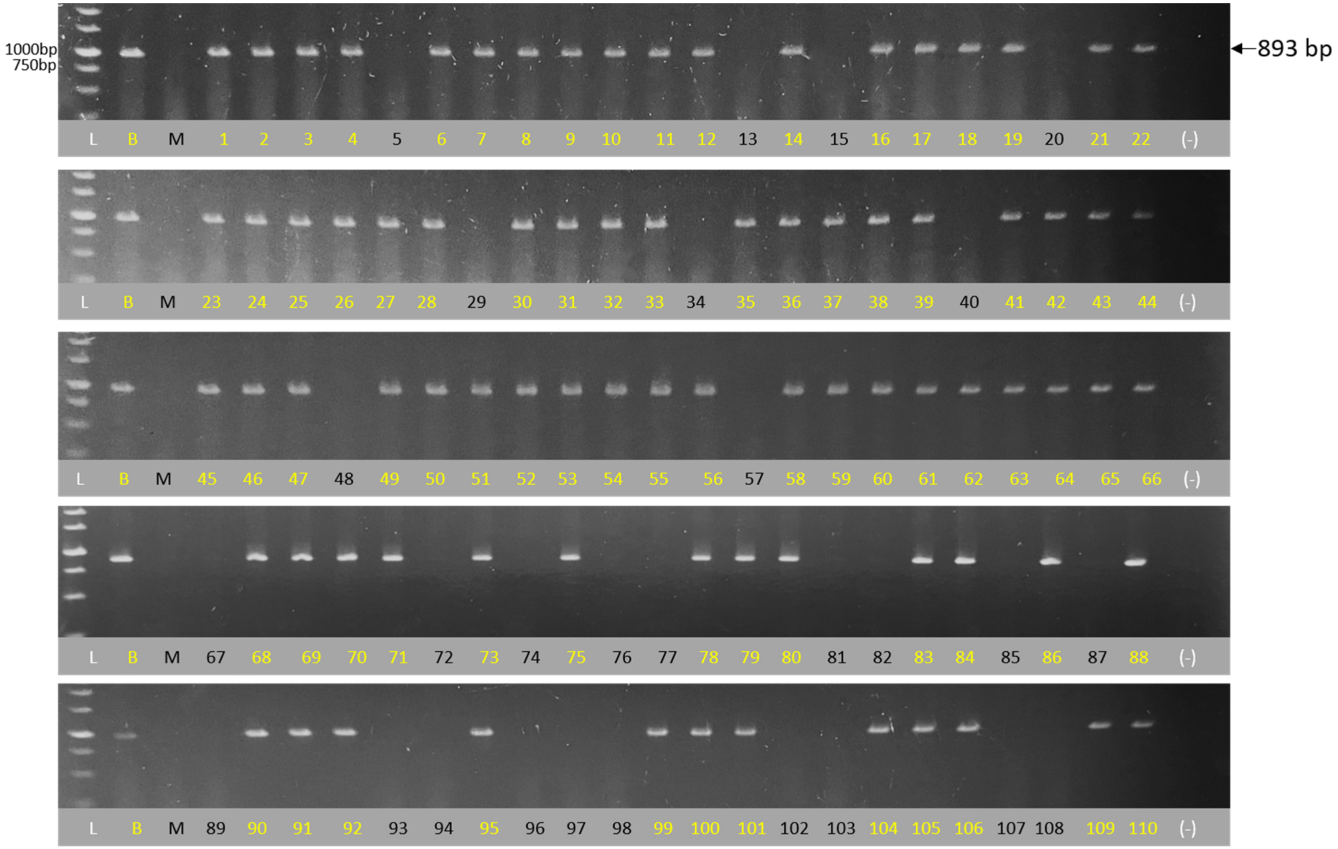

Figure S7. PCR genotyping of the F_2 population from DT26BS × DT26 using the Frag9 marker.

Total 223 individuals (numbered 1 to 223) in which 166 have wild-type yellow seeds and 57 have mutant black seeds. L: DNA ladder; B: wild-type; M: mutant; (-) water negative PCR control. Each numbers represents one individual, with font color to indicate phenotype: yellow font, yellow seeds; black font, black seeds.

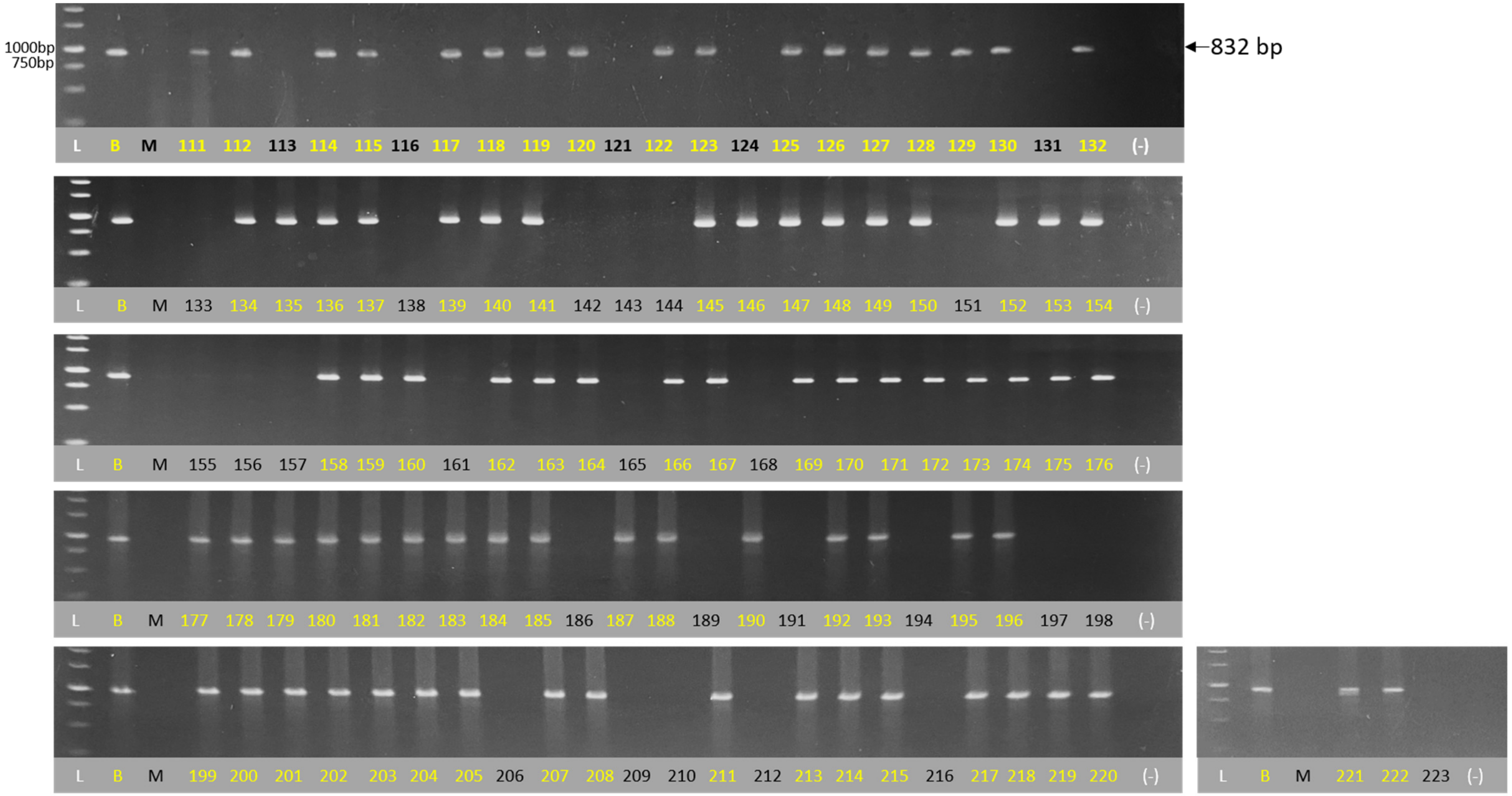

Figure S7 (continued). PCR genotyping of the F_2 population from DT26BS × DT26 using the Frag9 marker.

Total 223 individuals (numbered 1 to 223) in which 166 have wild-type yellow seeds and 57 have mutant black seeds. L: DNA ladder; B: wild-type; M: mutant; (-) water negative PCR control. Each numbers represents one individual, with font color to indicate phenotype: yellow font, yellow seeds; black font, black seeds.

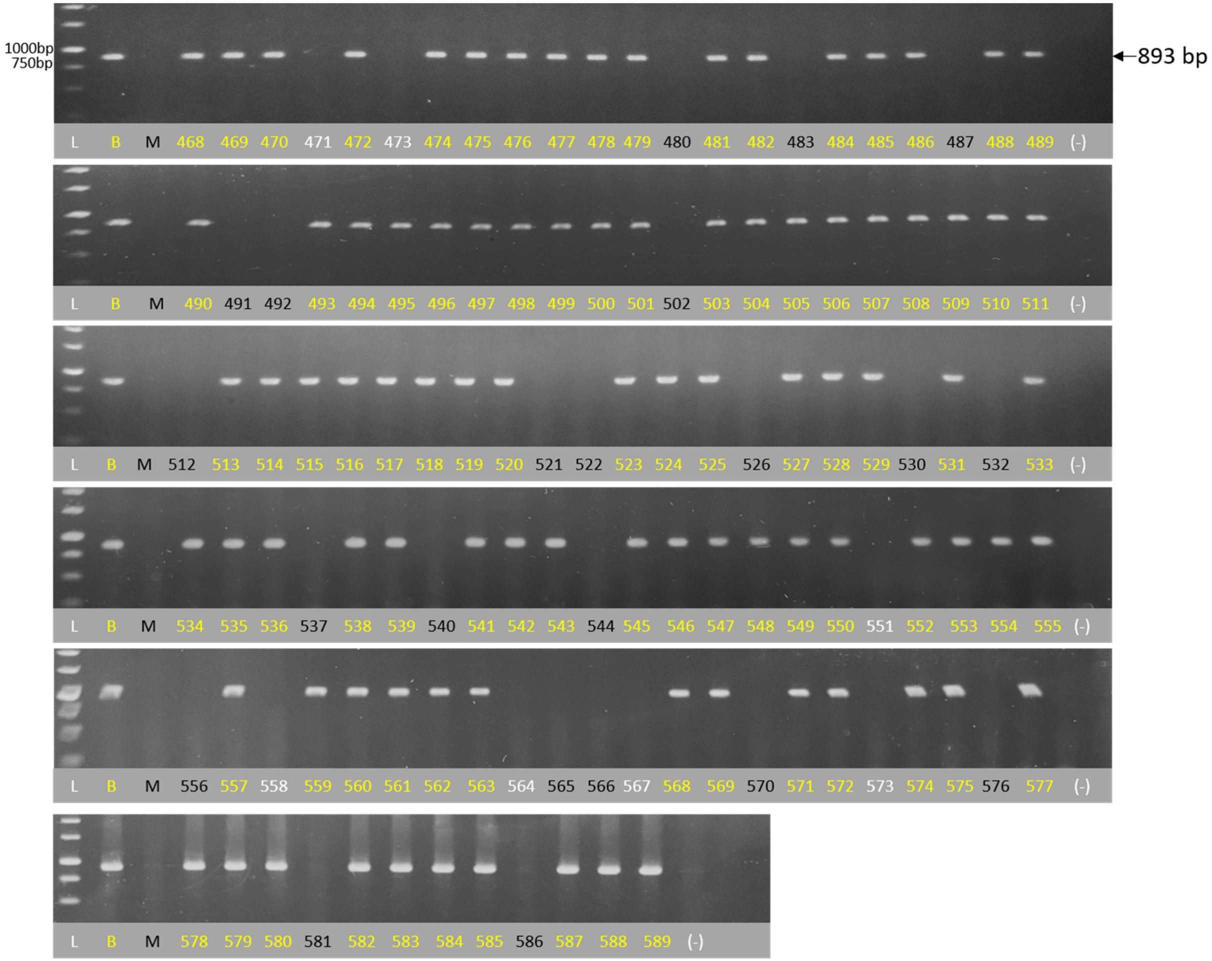

Figure S8. PCR genotyping of the F_2 population from DT84 × DT26BS-12 using the Frag9 marker.

A total of 122 individuals (numbered 468–589) were analyzed, including 93 plants with yellow seeds, 22 with black seeds, and 7 with brown seeds. L, DNA ladder; B, wild-type control; M, mutant control; (–), water negative control. Each number represents an individual, with font colour indicating phenotype (yellow font, yellow seeds; black font, black seeds; white font, brown seeds).

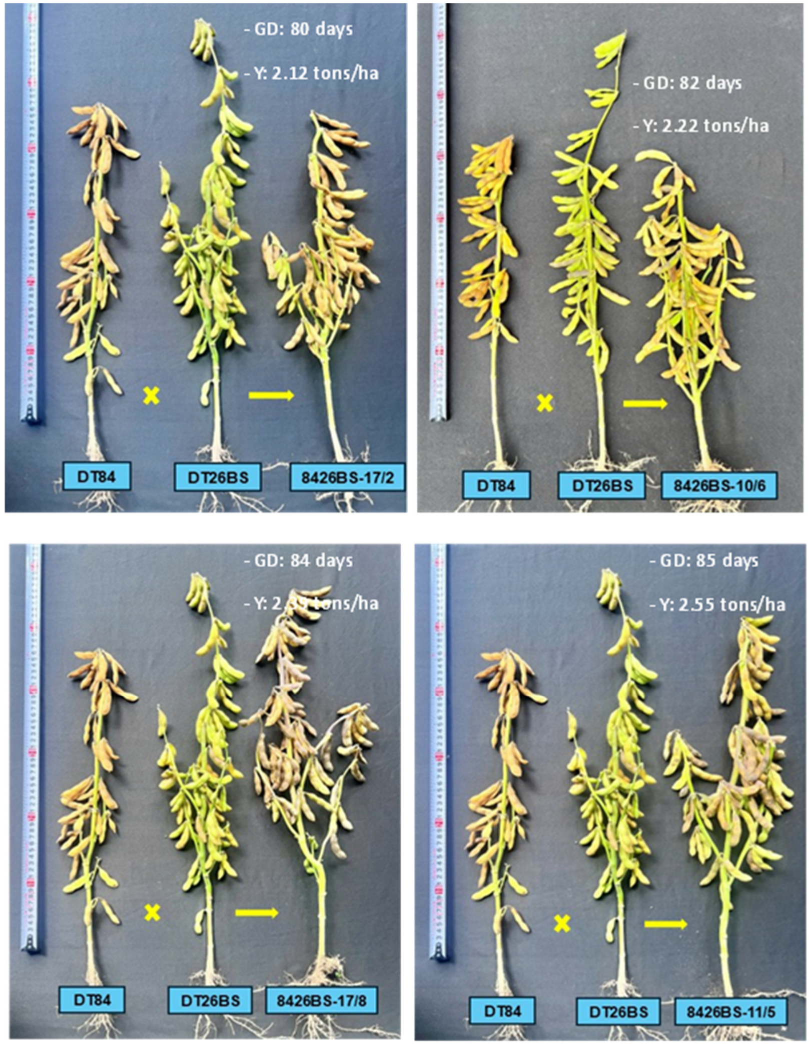

Figure S9. Development of new black soybean breeding lines in F7 generation. GD: Growth duration; Y: Yield

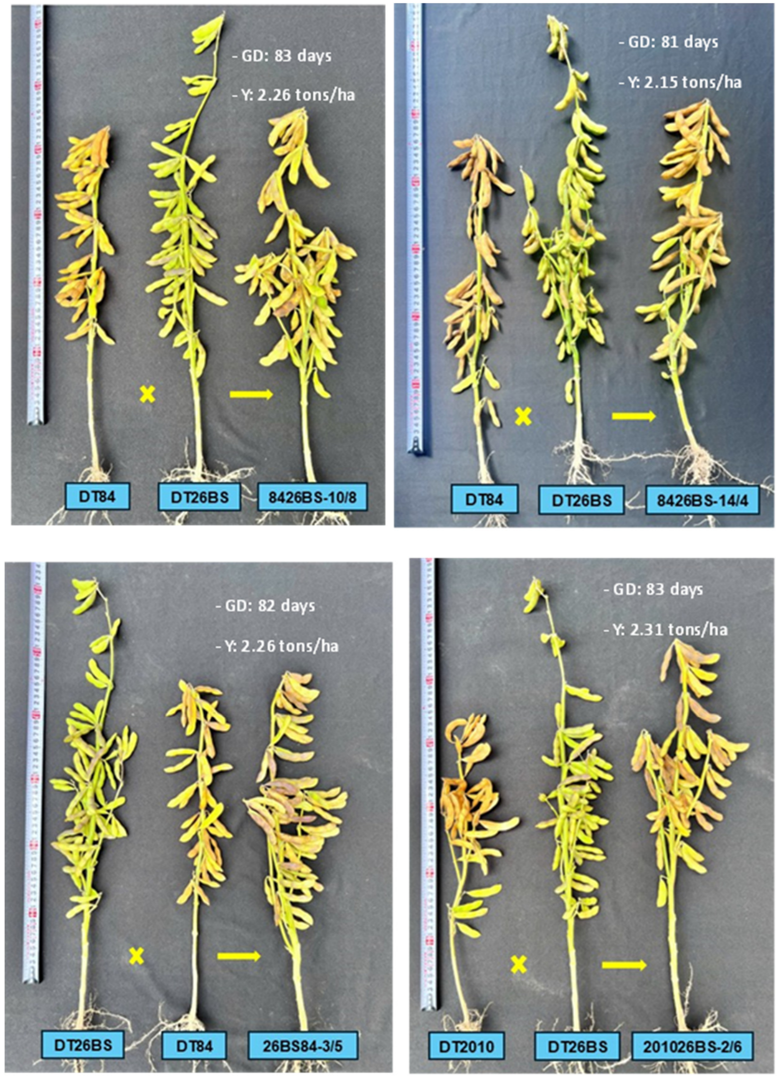

Figure S9 (continued)**.** Development of new black soybean breeding lines in F7 generation. GD: Growth duration; Y: Yield
