# Supplementary material for: MutMap Reveals a Structural Deletion at the Chalcone Synthase Locus Controlling Black Seed Coat in a Gamma-Irradiated Vietnamese Soybean Mutant
Source: Genes (Basel). 2026 Jul 17;17(7):814. doi: 10.3390/genes17070814 (PMC13409758; doi:10.3390/genes17070814)
Supplement: Supplementary file 1 [file genes-17-00814-s001.zip › Supplemental Table v3.pdf]

Table S1: Sequences of Primers used for validation of 12-bp deletion and regions locating CHS locus

Primer	Sequence (5′ to 3′)	Expected amplified Fragment
Del12bp-F	TCCAAGGTGAACCCGAATC	Del12bp marker
Del12bp-R	CGTCGTGTTAGGTGTACCT	
CHS4-40	CTTATATCCCACAACTTCTTAAC	Fragment 3
L813 (RC)	GGTGACGCTAGGGTTGAGGGAG	
CHS1-968	CTTACCCCCTCTACCAAACACACC	Fragment 9
Sa603/R140	GAATAAATGGAGCTTAGTTTG	

Table S2. Effect of different doses of radiation on survival and phenotypic mutant frequency

	M1 generation							M2 generation						
Radiation Dose (Gy)	0	100	150	200	250	300	350	0	100	150	200	250	300	**350**
Number of survival and screened individuals	482	481	458	422	342	226	101	25000	24700	20900	16500	8900	3200	500
Phenotypic	Number of individuals with mutant phenotype													
Sterility		1	5	14	27	32	20		272	266	239	238	188	39
Symmetrical branching				1							7	2		
Branching at cotyledon node			1	1	1	1				9	22	6		
Short stem (<5cm compared to check)			3	4	11	15	12				15	6		
High stem (>5cm compared to check)			1	1						5	3			
Abilism					1	1	1				3	9		
Early maturity (5 days shorter than check)										1	1			
Black seed coat										3				
Brown hillum										1				
More number of pods (> 20% compared to check)											3	3		
Curl stem										17	22	9		
Flat stem				1	1									
Double splitted stem			1								25	9		
Multi-branches (>2 branches compared to check)											2	3		
Non-branching										20		3		
Non-symmetrical branching											4			
Double splitted branches				1										
1-lobed leaflet				1	1									
2-lobed leaflet			1		1									

Table S3. Bio-agronomic characteristics and some nutrition content of DT26 and DT26BS

Traits	DT26	DT26BS
Flower color	White	White
Pubescence color	Brown	Brown
Pod color	Dark brown	Dark brown
Seed coat color	Yellow	Black
Hillum color	Dark brown	Black
Shape of lateral leaflet	Ovoid	Ovoid
Growth habit	Determinate	Determinate
Growth duration (days)	95-100	95-100
Stem height (cm)	40-60	40-60
Number of branches	2-4	2-4
Resistance to rust (1-9)*	3	3
Resistance to downy mildew (1-9)*	1	1
Resistance to powdery mildew (1-5)*	2	2
Resistance to brown spot (1-9)*	1-3	1-3
Tolerance to lodging (1-5)*	2	2
100-seed weight (g)	17-19	17-19
Yield (tons/ha)	2.0-2.5	2.0-2.5
Nutrition:		
Total anthocyanin content (mg/100g)	Not detected	42.45 ± 1.83
Total protein content (g/100g)	38.14 ± 0.03	38.83 ± 0.04
Total lipid content (g/100g)	20.48 ± 0.16	20.14 ± 0.09

*Scale provided in National Technical Regulation on Testing for Value of Cultivation and Use of Soybean varieties QCVN 01-58 : 2011/BNNPTNT, the smaller value the better the trait.

Table S4. Some characters of F7 lines with black seed coat derived from the combination DT84/DT26BS, DT26BS/DT84 and DT2010/DT26BS in Dan Phuong - Hanoi in winter 2025

Lines/Vars.	Growth duration (days)	Plant height (cm)	Number of branches	Number of 1-seed pods	Number of 3-seed pods	Total number of pods	100-seed weight (g)	Yield (tons/ha)
8426BS-17/2	80	40.6	3.1	3.4	9.9	41.1	18.7	2.12
8426BS-14/4	81	40.8	3.3	4.1	10.9	42.5	18.3	2.15
8426BS-10/6	82	39.2	4.1	3.3	10.9	43.8	18.1	2.22
8426BS-2/3	82	45.6	3.2	2.9	10.3	42.8	18.2	2.18
8426BS-7/4	82	40.4	3.4	4.8	12.9	43.7	18.0	2.21
8426BS-10/8	83	42.0	4.3	4.3	11.1	45.6	18.0	2.27
8426BS-10/9	83	65.1	4.2	3.7	13.9	44.6	17.8	2.26
8426BS-17/8	84	51.3	3.9	5.1	16.8	47.3	17.6	2.39
8426BS-6/7	84	43.3	4.6	5.5	15.1	45.8	18.3	2.37
8426BS-11/5	85	45.1	3.5	4.6	17.3	50.9	17.4	2.55
8426BS-14/9	85	71.5	4.1	2.7	14.7	47.7	17.8	2.45
26BS84-1/2	81	48.5	3.7	4.3	10.5	44.1	18.0	2.18
26BS84-3/5	82	46.4	4.1	4.7	11.5	45.2	18.0	2.26
26BS84-2/1	85	60.7	4.2	4.6	16.1	46.5	18.0	2.42
201026BS-2/2	80	39.7	4.3	3.1	10.3	36.4	20.2	2.07
201026BS-1/5	78	35.1	3.1	3.3	11.1	38.2	19.8	2.14
201026BS-2/6	83	55.1	4.3	4.5	10.2	42.1	20.1	2.31
DT84	80	40.2	2.2	2.5	10.3	36.9	18.7	1.95
DT2010	83	36.9	4.1	7.1	8.2	35.6	22.1	2.11
DT26BS	95	58.5	4.7	3.1	18.9	49.8	18.2	2.78
